# Comparing the Gene Expression Profile of Stromal Cells from Human Cord Blood and Bone Marrow: Lack of the Typical “Bone” Signature in Cord Blood Cells

**DOI:** 10.1155/2013/631984

**Published:** 2013-09-16

**Authors:** Julia Bosch, Amelie Pia Houben, Tatiana Hennicke, René Deenen, Karl Köhrer, Stefanie Liedtke, Gesine Kögler

**Affiliations:** ^1^Institute for Transplantation Diagnostics and Cell Therapeutics, Heinrich-Heine-University Medical Center Düsseldorf, Düsseldorf, Germany; ^2^Biological and Medical Research Center (BMFZ), Heinrich-Heine-University Düsseldorf, Düsseldorf, Germany

## Abstract

With regard to the bone-regenerative capacity, bone marrow stromal cells (BMSC) can still be termed the “gold standard.” Nevertheless, neonatal stromal cells from cord blood (CB) feature advantages concerning availability, immaturity, and proliferation potential. The detailed gene expression analysis and overexpression of genes expressed differentially provide insight into the inherent capacity of stromal cells. Microarray and qRT-PCR analyses revealed closely related gene expression patterns of two stromal cell populations derived from CB. In contrast to the CB-derived cell types, BMSC displayed high expression levels of *BSP*, *OSX*, *BMP4*, *OC*, and *PITX2*. Lentiviral overexpression of *BSP* but not of *OSX* in CB-cells increased the capacity to form a mineralized matrix. *BMP4* induced the secretion of proteoglycans during chondrogenic pellet culture and extended the osteogenic but reduced the adipogenic differentiation potential. BMSC revealed the typical osteogenic gene expression signature. In contrast, the CB-derived cell types exhibited a more immature gene expression profile and no predisposition towards skeletal development. The absence of *BSP* and *BMP4*—which were defined as potential key players affecting the differentiation potential—in neonatal stromal cells should be taken into consideration when choosing a cell source for tissue regeneration approaches.

## 1. Introduction 

With respect to the regeneration of cartilage or bone after tumor resection, accidents, or due to diseases affecting the skeleton, there is, great need for tissue-engineered bone. The cellular component of these approaches has been in the focus of interest for many years. 

The first described [1] and therefore the best studied nonhematopoietic stromal cell type derives from bone marrow (BM). The *in vivo* bone forming potential—including recruitment of hematopoietic cells of recipient origin—of these bone marrow stromal cells (BMSC) after transplantation on a hydroxyapatite scaffold was reported by several groups [2, 3]. The potential risks associated with the bone marrow donation made other sources of stromal cells, for example, adipose tissue or peripheral blood, attractive alternatives. Due to its immaturity compared to adult bone marrow, neonatal cord blood (CB), which can be collected noninvasively and without ethical concerns, can be regarded as a proper source of neonatal stromal cells with potential clinical relevance in the future. Cord blood contains at least two distinct populations of nonhematopoietic stromal cells with comparable proliferative potential [4], which were termed unrestricted somatic stromal cells (USSC) and cord blood-derived stromal cells (CBSC). So far, USSC and CBSC cannot be isolated prospectively but can be distinguished on the basis of cell surface antigens, differentiation potential, and gene expression. In flow cytometric analyses, CBSC revealed a stronger expression of CD146 (MCAM, melanoma adhesion molecule) compared to USSC [4]. During *in vitro* differentiation assays, CBSC but not USSC possess the potential to differentiate into adipocytes [5]. Former results indicated a correlation of the absent adipogenic potential and the expression of* DLK1* (delta, *Drosophila* homolog-like 1) in USSC, since USSC but not CBSC express *DLK1* [5]. Recent results suggested that *DLK1* might not be the sole factor responsible for the inhibition of *in vitro* adipogenesis in USSC [6]. In microarray and PCR analyses, the expression of *HOX *(homeobox) genes was defined as additional distinguishing feature: USSC completely lack *HOX* gene expression, while CBSC are *HOX* positive [7]. Furthermore, USSC can be discriminated from CBSC on the basis of their higher hematopoiesis-supporting capacity in coculture experiments [6].

To date, the proof of the ability of CB-derived stromal cells to form true bone and to recruit hematopoietic cells after transplantation in standardized *in vivo* assays is still missing. Before performing such assays, the identification of potential differences on molecular level between CB-cells and the “gold standard” BMSC is mandatory. With respect to their immunophenotype, CB- and BM-derived cells are barely different. A potential cell surface marker to distinguish these cell types quantitatively by flow cytometric analyses is CD146 [4], but this antigen was also described to be expressed on pericytes, regardless if they are osteogenic or not [3]. On transcriptome level, differences in the gene expression were described for cell types of distinct origin [8]. In the present study, further genes expressed differentially in BM- and CB-derived cell populations were examined to find potential candidate genes influencing the *in vivo* regenerative potential. Special attention was paid to genes regulating the formation of the skeleton by endochondral or intramembranous ossification during fetal development. 

Chondrogenesis is precisely adjusted by extracellular matrix and growth factor signals as well as by intracellular signaling pathways and gene transcription in a temporal-spatial manner [9]. Essential regulatory pathways involved in fetal chondrogenesis are FGF, hedgehog, BMP, or WNT signaling [9, 10]. BMPs—in particular *BMP2*, *4*, and *7*—are known to act during early (chondroprogenitor cell determination and differentiation) and late stages (terminal differentiation to hypertrophic chondrocytes) of chondrocyte maturation [9]. Furthermore, *BMP4* is also involved in the regulation of osteoblast maturation [11]. During endochondral ossification, the cartilaginous matrix is replaced by bone matrix synthesized by osteoblasts. One of the most important and earliest transcription factors controlling this process is the runt-related transcription factor 2 (*RUNX2*) which, for instance, binds the promotor of the osteoblast-specific hormone osteocalcin (*OC*) [12]. Beside *RUNX2*, the transcription factor SP7 (osterix, *OSX*) is essential for the differentiation of osteoblasts in mice: inactivation of *Osx* leads to a failure in bone formation [12, 13]. *OSX* is located downstream of *RUNX2*, as evidenced by its absence in *Runx2*-deficient mice [13]. In the later stages of bone formation, the newly formed bone matrix mineralizes through accumulation of hydroxyapatite, collagens, and noncollagenous proteins. The secreted phosphoprotein *BSP* (integrin-binding sialoprotein) constitutes the main part of the noncollagenous proteins of the human bone extracellular matrix [14]. An essential role for *Bsp* regarding the *in vivo* bone forming potential has been reported for murine BMSC: only clonal cell lines expressing *Bsp* revealed an *in vivo* osteogenic potential, whereas the *Bsp*-negative cell lines were nonosteogenic [15].

In the present study, genes differentially expressed in stromal cells from cord blood (USSC and CBSC) and bone marrow (BMSC), which potentially affect the *in vivo* bone forming capacity, were identified by microarray data analyses and quantitative RT-PCR. *BMP4*, *BSP*, and *OSX* were stronger expressed in BM- compared to CB-derived stromal cells and were selected for overexpression experiments to assess the gene function during the regulation of differentiation. Further analyses indicated an osteosupportive role for *BMP4* and *BSP*, whereas *OSX* seemed to have a negative effect on the bone forming capacity *in vitro*.

## 2. Materials and Methods 

### 2.1. Isolation and Expansion

The ethical review board of the Medical Faculty of the University Düsseldorf granted the ethical approval to isolate the different cell types (Study nos. USSC/CBSC: no. 2975, BMSC: no. 3240).

USSC and CBSC were isolated using the same protocol. To discriminate the cell types, the adipogenic differentiation potential as well as the *DLK-1* [5] and *HOX* gene expression [7] was determined in passage 4 or 5. The immunophenotype and growth potential of both cell types were compared in a previous study [4].

The cell isolation was conducted as published before [5, 16]. In brief, human CB was collected from the umbilical cord vein with written informed consent of the mothers. The mononuclear cell fraction (MNC) was obtained by ficoll gradient separation (Biochrom AG) followed by ammonium chloride lysis of red blood cells. 5–7 × 10^6^ MNC/mL were cultured in Dulbecco's Modified Eagle Medium (DMEM) low glucose (Lonza) with 30% fetal calf serum (FCS, Hyclone), 10^−7 ^M dexamethasone (Sigma-Aldrich), and 1% penicillin/streptomycin/Lglutamine (PSG, Lonza). Single colonies were detached with trypsin (0.25%) using cloning cylinders (Merck Millipore) and expanded in the same medium without dexamethasone. 

BMSC were isolated using bone marrow aspirated from the iliac crest of healthy donors as described previously [17]. 

All cell types were cultured at 37°C in a humidified atmosphere with 5% CO_2_ until reaching 80% confluence. USSC and CBSC were detached with 0.25% trypsin, while BMSC were detached with 0.25% trypsin/EDTA (both Lonza). 

### 2.2. Microarray Gene Expression Analyses

Cell lines in passage 5 were used for microarray gene expression analyses. Total RNA was extracted according to the RNeasy Mini Kit protocol (Qiagen). RNA preparations were checked for RNA integrity by Agilent 2100 Bioanalyzer. All samples in this study showed high-quality RNA integrity numbers (RINs) of 10. RNA was quantified by photometric Nanodrop measurement. Synthesis of cDNA and subsequent biotin labeling of cRNA was performed according to the manufacturers' protocol (3' IVT Express Kit; Affymetrix, Inc.). Briefly, 100 ng of total RNA was converted to cDNA, followed by *in vitro* transcription and biotin labeling of aRNA. After fragmentation, labeled aRNA was hybridized to Affymetrix PrimeView Human Gene Expression Microarrays for 16 h at 45°C, stained by streptavidin/phycoerythrin conjugate, and scanned as described in the manufacturers' protocol. Data analyses on digitized fluorescence signal intensities were conducted with GeneSpring GX software (Vers. 12.1; Agilent Technologies). Probes within each probeset were summarized by RMA after quantile normalization of probe level signal intensities across all samples to reduce interarray variability [18]. Input data before processing was concluded by baseline transformation to the median of all samples. After grouping of samples according to their respective experimental conditions (USSC, CBSCs and BMSC, three replicates each), a given probeset had to be expressed above background (i.e., fluorescence signal of a given probeset was detected within the 20th and 100th percentiles of the raw signal distribution of a given array) in at least two of the three replicates in every single one of the three experimental groups. The resulting cell-type-specific gene expression profiles was compared by separating overlapping from cell type specific gene lists (Venn diagram analysis). Global similarity of gene expression profiles were determined by principal component analysis (PCA). Expression values were mean centered and scaled to unit standard deviation. Pruning options within GeneSpring GX software were set to a fixed number of principal components (numPrincipalComponents = 3).

Those genes expressed uniquely in one cell population (Venn diagram) were grouped using the “Functional Annotation Cluster Tool” provided by DAVID Bioinformatics Resources (http://david.abcc.ncifcrf.gov/) [19–21].

### 2.3. Quantitative Reverse Transcription Polymerase Chain Reaction (qRT-PCR)

RNA isolation was performed using RNeasy Kits (Qiagen). RNA of differentiated cells was isolated using Tri Reagent (Sigma-Aldrich) according to the manufacturer's instructions followed by DNA digestion with DNase I (Life Technologies). Prior to RNA isolation, chondrogenic pellets were incubated at 37°C in pronase E (Merck) for 1 h followed by incubation in collagenase P (Roche) for 24 h. Reverse transcription (RT) was performed with SuperScriptIII (Life Technologies) according to the supplier's protocol. Complementary DNA (cDNA) which approximated 50 ng of RNA was used for subsequent qRT-PCR with Power SYBR Green PCR Mastermix (Life Technologies). The primer sequences and corresponding annealing temperatures are listed in Table S1 available online at http://dx.doi.org/10.1155/2013/631984. To screen the genes associated with the *WNT* pathway, WNT signaling pathway PCR arrays (PAHS-04, SA Biosciences, Qiagen) were applied (the gene expressions of *MYC* and *PITX2* are presented in this paper). After comparison of different potential housekeeping genes regarding the gene expression stability during the differentiation process (data not shown), we decided to use human ribosomal protein L13a (*RPL13A*) as reference gene for normalization. For *SOX9*, a TaqMan Gene Expression Assay in combination with the TaqMan 2x Universal PCR Master Mix No Amp Erase UNG (all Life Technologies) was used. All reactions were run in duplicates on an ABI Step One Plus Detection System using the following standard program: 95°C 10 min; 95°C 15 s, 55/60/65°C 1 min (40 cycles). Relative changes in gene expression were calculated following the ΔΔCt-method.

### 2.4. Statistical Analysis

Data are presented as arithmetic means with standard deviation of at least three different cell lines. Unpaired *t*-tests were conducted with GraphPad Prism Version 5.01. *P* values lower than 0.05 were considered significant (*P = 0.01 to 0.05; **P = 0.001 to 0.01; ***P < 0.001).

### 2.5. *In Vitro* Differentiation

For induction of adipogenic differentiation, cells were plated at 8.3 × 10^3^ cells/cm^2^ in 6-well plates until reaching 70% confluence. Adipogenic differentiation media were changed twice a week for 21 days, alternating induction and cultivation medium. The former consisted of DMEM high glucose (Lonza) supplemented with 10% FCS, 1% penicillin/streptomycin/Lglutamine (PSG), 10^−6 ^M dexamethasone, 0.2 mM indomethacin, 0.1 mg/mL insulin, and 1 mM 3-isobutylmethylxanthine (all Sigma-Aldrich); the latter was made up of DMEM high glucose, 10% FCS, 1% PSG, and 0.01 mg/mL insulin. As a negative control, the cells were cultured in DMEM low glucose, 10% FCS, and PSG. The differentiated cells were fixed with formaldehyde and stained with Oil Red O (Sigma-Aldrich) to visualize lipid vacuoles. The stained lipid vacuoles were quantified using the ImageJ Java-based image processing software for Windows. A minimum of 3 pictures for each experiment were analyzed. The stained area was calculated and the corresponding negative control was subtracted.

For induction of osteogenic differentiation, cells were plated at 8.3 × 10^3^ cells/cm^2^ in 6-well plates. When reaching 70% confluence, the osteogenic differentiation medium containing DMEM low glucose supplemented with 30% FCS, 1% PSG, 10^−7^ M dexamethasone, 50 *μ*g/mL ascorbic acid, and 10 mM beta-glycerolphosphate (all Sigma-Aldrich) was added. As a negative control, the cells were cultured in DMEM low glucose, 10% FCS, and PSG. Osteogenic differentiation was performed for 14 days; the medium was changed twice a week. To detect mineralization, a staining with silver nitrate (“Von Kossa”) or Alizarin Red S applying standard protocols was performed. For Von Kossa staining, the cells were fixed in cold ethanol (70%, 10 min), incubated in silver nitrate (Roth, 5%, 30 min) followed by sodium thiosulfate pentahydrate (Merck, 1%, 1 min). Nuclear fast red aluminium sulfate solution (Merck, 0.1%, 30 min) was applied for counterstaining. Distilled water was used to wash the cells between the steps of the staining procedure. For Alizarin Red S-staining, the fixation was performed in cold ethanol (70%, 10 min), followed by incubation in Alizarin Red S (Sigma-Aldrich, 2%, 10 min) and 5 washing steps with distilled water. After the staining procedure, the amount of Alizarin Red was quantified. 800 *μ*L of acetic acid was added and incubated for 30 min under permanent shaking. The cell layer was detached with a cell scraper, vortexed, and incubated first at 85°C for 10 min, then on ice for 5 min. After a centrifugation step (24500 g, 15 min), 500 *μ*L of the supernatant was mixed with 200 *μ*L of ammonium hydroxide (10%) and was analyzed photometrically (plate reader, Bio-Tek Instruments Inc.) at 405 nm. Values of the negative control were subtracted from those of differentiated cells. Each sample was measured in triplicates.

To induce chondrogenesis, aliquots of 2 × 10^5^ cells were centrifuged at 150 g for 7 min in 15 mL polypropylene conical tubes. The pelleted cells were incubated for 21 days in DMEM high glucose supplemented with 1% PS, 100 nM dexamethasone, 35 *μ*g/mL ascorbic acid-2-phosphate, 1 mM sodium pyruvate (all Sigma-Aldrich), Insulin-Transferrin-Selenium (1/100 dilution) (Gibco), and 10 ng/mL TGF beta1 (MACS, Miltenyi Biotec). The media were changed three times a week. For Safranin O/Fast Green staining, the pellets were embedded in Tissue Freezing Medium (Jung, Leica) and cut into sections of 6 *μ*m using a cryotome. The slides were fixed with cold ethanol (70%), stained with Safranin O for 30 min and Fast Green (both Waldeck) for 5 s. After washing in distilled water, the slides were incubated in ethanol (96%) and xylol. Entellan (Merck) was used as mounting medium. 

### 2.6. Lentiviral Overexpression

To isolate DNA containing the gene of interest, specific primers with restriction sites for the restriction enzymes were designed (Table S1). The genes fragments were inserted into the pCL6IEGwo vector (Figures S1 and S2). *E. coli* TOP 10 (Life Technologies) was used for transformation. To verify correct gene delivery, the constructs (pCL6*BMP4*, pCL6*BSP*, and pCL6*OSX*) were sequenced using the BigDye Terminator Cycle Sequencing Kit (Life Technologies). Lentiviral particles were produced using FuGENE transfection reagent (Roche) to transfect HEK293T cells with the envelope plasmid pALF-GALV, the helper plasmid pCD/NL-BH, and the expression vector pCL6IEGwo containing eGFP and the cloned gene sequence (Figure S2). HEK293T transfection was accomplished according to the following protocol. Day 1: HEK293T were plated in DMEM (high glucose), 10% FCS, and 1% PSG (5 × 10^5^ cells/cm^2^) on 10 cm plates. Day 2: HEK293T transfection: DMEM (high glucose), 5 *μ*g of each plasmid, and 45 *μ*L FuGENE were mixed and incubated for 15 min at room temperature and added to HEK293T in DMEM (high glucose), 5% FCS. Day 3: target cells were plated (1 × 10^5^ cells/60 cm^2^). HEK293T culture medium was changed (DMEM (high glucose), 5% FCS, 1% PSG). Day 4: infection of target cells: HEK293T supernatant containing virus particles was sterile filtered (0.45 *μ*m filter) and added to target cells (diluted if necessary). For control cells (“Mock”), medium without virus particles (DMEM (high glucose), 5% FCS, 1% PSG) was applied. Days 5 and 6: medium change of target cells (DMEM (low glucose), 30% FCS, and 1% PSG). To ensure a high transfection efficiency of the target cells, the eGFP expression was measured via flowcytometric analysis. Fluorescence-activated cell sorting (FACS) was accomplished in the Core Flow Cytometry Facility of the Heinrich-Heine-University Medical Center Düsseldorf, Germany. 

## 3. Results

### 3.1. The Gene Expression Profiles of Neonatal USSC and CBSC Are More Similar to Each Other Than to Adult BMSC

**Figure 1:**
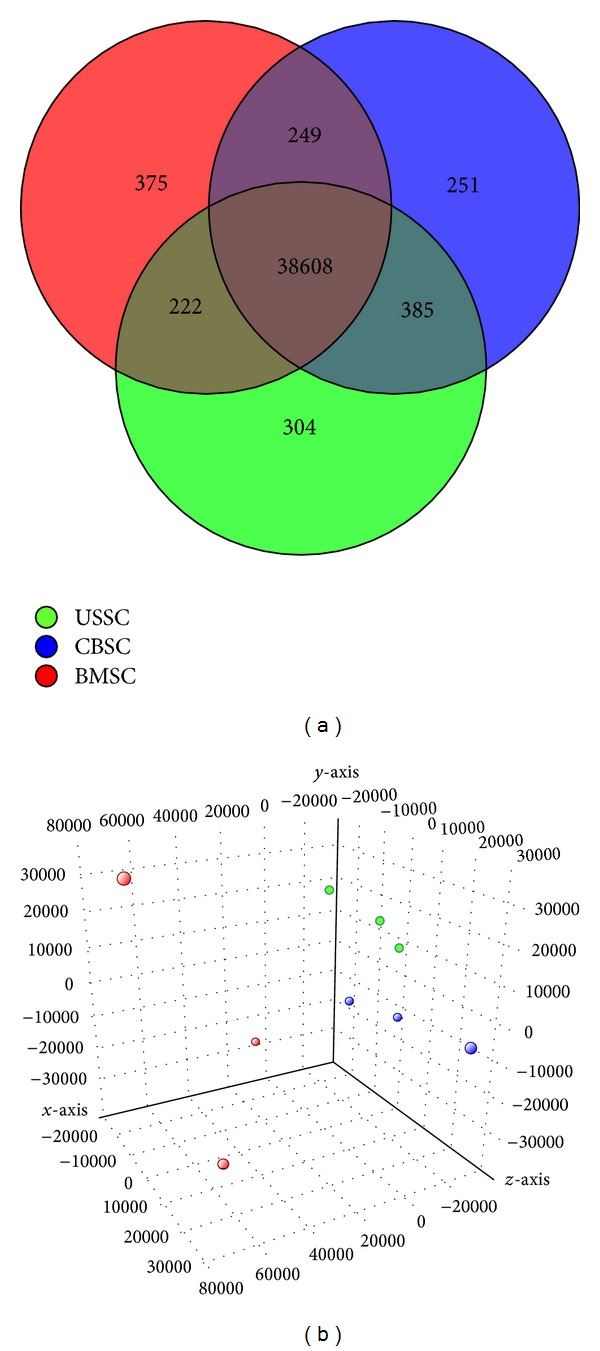
PrimeView human gene expression array. The gene expression of USSC (green), CBSC (blue), and BMSC (red, each in triplicate) was analyzed. (a) Venn-diagram illustrating common and unique expression of genes. The gene expression was filtered for each cell type: a probeset had to be expressed above the background (20th and 100th percentiles of the raw signal distribution) in at least two out of three replicates. This resulted in 39519 transcripts for the “group USSC,” 39493 transcripts for CBSC, and 39454 transcripts for BMSC which were compared. (b) principal component analysis (PCA) to depict the correlation of the single replicates of each cell type. Analyzed were those probesets that were expressed above the background in at least two out of three replicates in at least one out of three cell types which resulted in 40394 transcripts. The first three principal components accounted for 62.9% of the total variance (component 1 (*x*-axis): 31.6%; component 2 (*y*-axis): 18.3%; component 3 (*z*-axis): 13.0%).

To get an overview of the gene expression profiles of USSC, CBSC, and BMSC (three replicates each), microarray gene expression analyses were performed. The results were depicted in a Venn diagram and a principal component analysis (PCA, Figures 1(a) and 1(b)). 

The Venn diagram illustrates the count of genes expressed by one or by more cell types. The vast majority of genes was expressed in common by all three cell types (38608 genes, Figure 1(a)). Among those genes not expressed by all cell types, USSC and CBSC expressed 385 genes which were absent in BMSC. 249 genes were present in CBSC and BMSC but not in USSC, while the expression of 222 genes was shared by USSC and BMSC. 304 genes were expressed uniquely in USSC, 251 in CBSC and 375 in BMSC (Figure 1(a)). For detailed analyses, those genes were assigned to biological functions (gene ontology (GO) terms) using the functional annotation cluster tool of the DAVID Bioinformatics Resources website. In Table 1, those genes expressed uniquely in USSC, CBSC, or BMSC associated with the process of osteogenesis are listed. USSC and CBSC exhibited the unique expression of only three “bone-related” genes in three GO terms each. On the contrary, BMSC expressed 13 genes grouped in ten GO terms that the CB-derived cell types did not express (Table 1). 

The principal component analysis presents the correlation between the three replicates of one cell type depicted as spheres in a three-dimensional space. Those spheres which display a high gene expression similarity are positioned closer to each other. The analysis of the three bone marrow cell lines revealed a scattering in the three-dimensional space, whereas the triplicates of USSC and CBSC are closer related to each other (Figure 1(b)). 

To summarize, the analysis of the microarray data suggests a more similar gene expression pattern of USSC and CBSC compared to that of BMSC (Figure 1(a)). In addition, BMSC expressed more osteogenesis-related genes in unique than USSC or CBSC, indicating an inherent “osteogenic signature” of the bone-marrow-derived cells (Table 1). Furthermore, the BMSC cell lines exhibited a stronger biological variance compared to USSC or CBSC cell lines (Figure 1(b)).

### 3.2. Differentially Expressed Genes Were Evaluated by Quantitative RT-PCR: BMSC Exhibited an Osteogenic Signature

**Figure 2:**
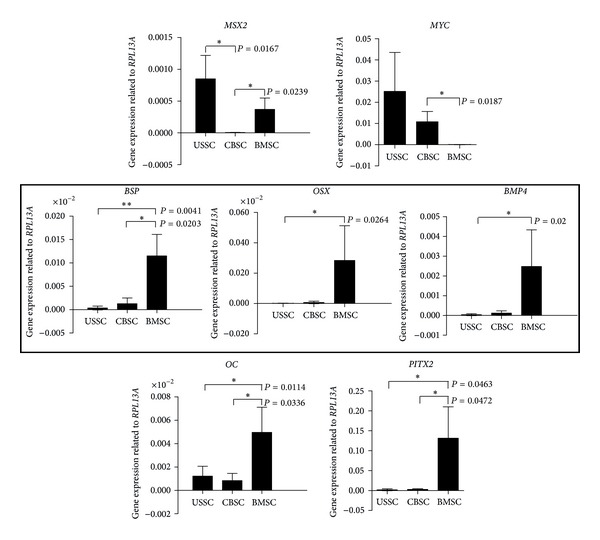
Quantitative RT-PCR analyses of genes expressed differentially in USSC, CBSC, and BMSC. Illustrated are the arithmetic means and standard deviations of at least 3 different cell lines per cell type. *P = 0.01 to 0.05, significant; **P = 0.001 to 0.01, very significant (unpaired *t*-test). *RPL13A* was used as housekeeping gene. Genes selected for overexpression experiments are highlighted. MSX2: homolog of muscle segment homeobox Drosophila 2, MYC: V-Myc avian myelocytomatosis viral oncogene homolog, BSP: integrin-binding sialoprotein, OSX (SP7): osterix, BMP4: bone morphogenetic protein 4, OC (BGLAP): osteocalcin, PITX2: paired-like homeodomain transcription factor 2, and RPL13A: ribosomal protein L13A.

After genome wide microarray analyses, a more detailed insight was gained by qRT-PCRs to assess genes expressed differentially in the cell populations. After interpretation of the microarray gene expression data, special focus was placed on genes associated with the process of bone formation. The gene expression was analyzed in at least three different cell lines per cell type to compensate the biological variance. Integrin-binding sialoprotein (*BSP*), osterix (*OSX*), bone morphogenetic protein 4 (*BMP4*), osteocalcin (*OC*), and paired-like homeodomain transcription factor 2 (*PITX2*) revealed a stronger expression in BMSC compared to the CB-derived cell types (Figure 2, unpaired *t*-test: *BSP*: USSC/BMSC, *P* = 0.004, CBSC/BMSC, *P* = 0.02; *OSX*: USSC/BMSC, *P* = 0.03, CBSC/BMSC, not significant (n.s.); *BMP4*: USSC/BMSC, *P* = 0.02, CBSC/BMSC, n.s.; *OC*: USSC/BMSC, *P* = 0.01, CBSC/BMSC, *P* = 0.03; *PITX2*: USSC/BMSC, *P* = 0.046, CBSC/BMSC *P* = 0.047). Homolog of muscle segment homeobox *Drosophila* 2 (*MSX2*) was stronger expressed in USSC and BMSC, whereas CBSC revealed a reduced gene expression (Figure 2, unpaired *t*-test: USSC/CBSC, *P* = 0.02; CBSC/BMSC, *P* = 0.02). The qRT-PCR analysis of V-Myc avian myelocytomatosis viral oncogene homolog (*MYC*) revealed the strongest expression in USSC followed by CBSC. BMSC expressed *MYC* only slightly (Figure 2, unpaired *t*-test: USSC/BMSC, n.s.; CBSC/BMSC, *P* = 0.02).

BMSC showed a high expression level of the osteogenesis-related genes *BSP*, *OSX,* and *BMP4. *Taken into account that bone-marrow-derived stromal cells can still be denoted as the most reliable source for *in vivo* bone regeneration [22], these genes are potential candidate genes for the regulation of the osteogenic potential of a cell type. Thus, overexpression experiments of these genes were performed to assess the gene function in neonatal stromal cells.

### 3.3. Overexpression of *BSP* Resulted in Increased Calcification

**Figure 3:**
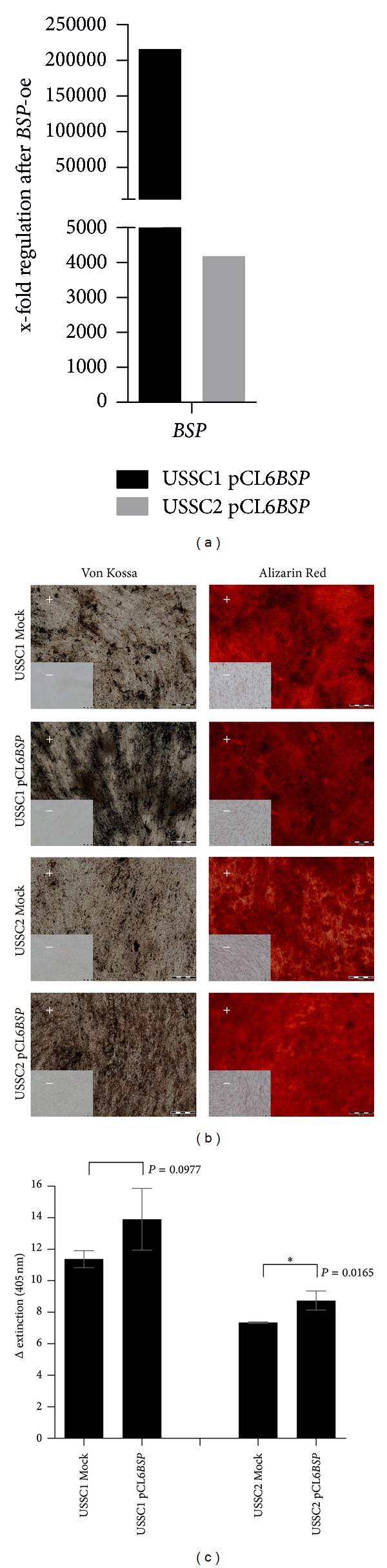
Lentiviral overexpression of *BSP* in two USSC cell lines. (a) Quantitative RT-PCR analysis of *BSP* gene expression (in undifferentiated cells) after overexpression in relation to the control cells (Mock). *RPL13A* was used as housekeeping gene. (b) Osteogenic differentiation experiments in overexpressed and Mock cells. After 14 days of differentiation, Von Kossa and Alizarin Red S staining were performed in the induced (+) and noninduced (−) cells. Mineralized areas are stained in brown/black or red, respectively. Scale bar: 200 *μ*m. The results were confirmed in independent experiments; representative histological staining and the subsequent quantification of the bound Alizarin Red S-dye (c) are depicted. *P = 0.01 to 0.05, significant (unpaired *t*-test). *BSP*: integrin-binding sialoprotein *RPL13A*: ribosomal protein L13A.

With regard to the distinct expression pattern of *BSP* in CB-derived cell types compared to BMSC (Figure 2), lentiviral overexpression experiments were performed in two *BSP*-negative USSC cell lines. The transfection efficiency was proved via qRT-PCR (Figure 3(a), USSC1: 215927-fold stronger expression in relation to Mock control-cells, USSC2: 4185-fold stronger). The potential to differentiate towards adipocytes was not affected by the overexpression (data not shown). In contrast, the osteogenic *in vitro* differentiation potential was improved after *BSP *overexpression as analyzed by Von Kossa and Alizarin Red S staining (Figure 3(b)) with subsequent quantification (Figure 3(c)). Von Kossa staining revealed an intensified brown/black staining due to stronger calcification after overexpression of *BSP* compared to the nontransfected cells (Mock). The light microscopic photos of Alizarin Red S-stained cells after differentiation did not allow an interpretation of the amount of calcification; therefore, the quantity of bound Alizarin Red S dye was measured (Figure 3(c), USSC1: *P* = 0.0977, n.s.; USSC2: *P* = 0.0165, significant).

Overexpression of *BSP* supported the *in vitro* osteogenic differentiation of both USSC cell lines transfected. Thus, the expression of *BSP* should be taken into consideration when assessing the osteogenic potential of a cell type. 

### 3.4. Overexpression of *OSX* Led to Decreased Osteogenic Differentiation Potential

**Figure 4:**
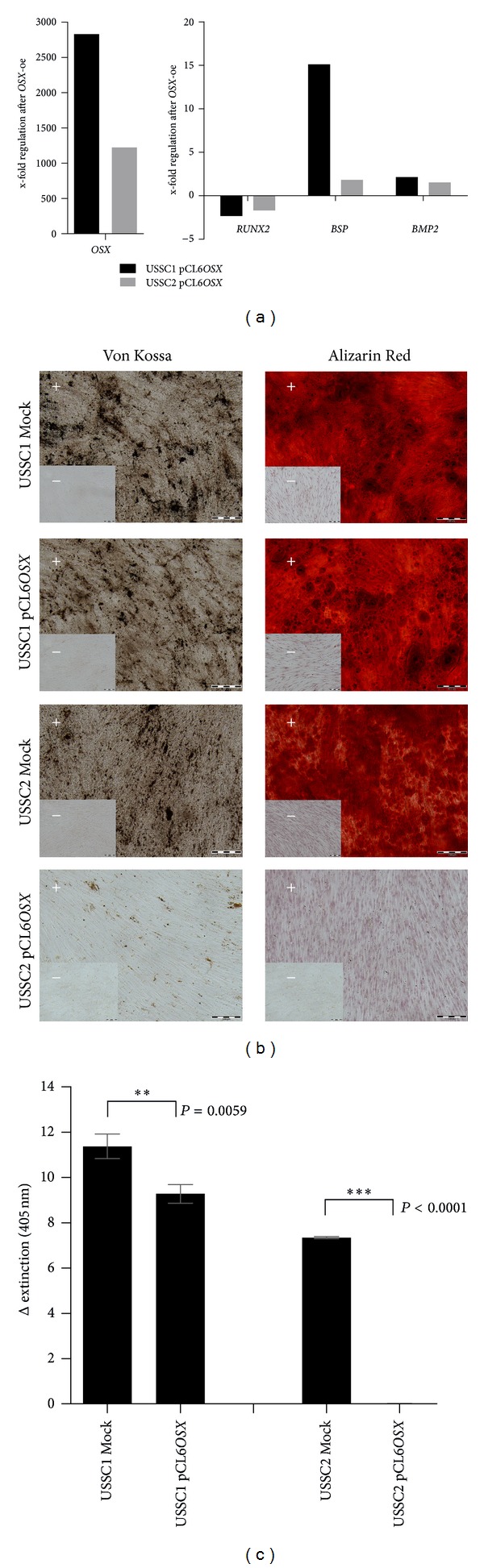
Lentiviral overexpression of *OSX* in two USSC cell lines. (a) Quantitative RT-PCR analysis of gene expression (in undifferentiated cells) after overexpression in relation to the control cells (Mock). *RPL13A* was used as housekeeping gene. (b) Osteogenic differentiation experiments in overexpressed and Mock cells. After 14 days of differentiation, Von Kossa and Alizarin Red S staining were performed in the induced (+) and non-induced (−) cells. Mineralized areas are stained in brown/black or red, respectively. Scale bar: 200 *μ*m. The results were confirmed in independent experiments; representative histological staining and the subsequent quantification of the bound Alizarin Red S-dye (c) are depicted. **P = 0.001 to 0.005, very significant; ***P < 0,001, extremely significant (unpaired *t*-test). *OSX (SP7)*: osterix, *RUNX2*: runt-related transcription factor 2, *BSP*: integrin-binding sialoprotein, *BMP2*: bone morphogenetic protein 2, and *RPL13A*: ribosomal protein L13A.

Comparable to the expression of *BSP*, *OSX* was almost absent in USSC while a minimal expression was detected in CBSC (Figure 2). Thus, overexpression of *OSX* was performed in two USSC cell lines.  Figure 4(a) illustrates the gene expression after *OSX* transfection (USSC1 Mock/pCL6*OSX*: 2831-fold increased expression, USSC2 Mock/pCL6*OSX: *1223-fold). Due to the *OSX* overexpression, the expression of *RUNX2* (runt-related transcription factor 2) was down regulated (Figure 4(a), USSC1: −2.3-fold compared to Mock cells, USSC2: −1.7-fold), whereas the expression of *BSP* and *BMP2* (bone morphogenetic protein 2) increased (Figure 4(a), *BSP*: USSC1: 15.1-fold increased expression compared to Mock cells, USSC2: 1.8-fold; *BMP2*: USSC1: 2.1-fold, USSC2: 1.5-fold). The adipogenic differentiation potential was not affected by the overexpression of *OSX* (data not shown). In contrast, the mineralization during osteogenic differentiation was reduced in *OSX*-transfected USSC which was analyzed using Von Kossa and Alizarin Red S staining and quantification (Figures 4(b) and 4(c)). Von Kossa staining revealed a slight decrease of calcification in the cell line USSC1 after overexpression of *OSX* which was confirmed by quantification of the bound Alizarin Red S dye (Figures 4(b) and 4(c), *P* = 0.0059, very significant). A more pronounced decrease of calcification after overexpression was detected in the cell line USSC2. In Von Kossa as well as in Alizarin Red S staining, almost no calcification was measured which was confirmed by Alizarin Red S quantification (Figures 4(b) and 4(c), *P* < 0.0001, extremely significant).

*OSX* was described to be essential for bone formation [13]. Nevertheless, in the present study a reduced *in vitro* osteogenic capability of USSC after *OSX* overexpression associated with a decreased expression of *RUNX2* was detected. The upregulation of *BSP* and *BMP2* after overexpression did not support the osteogenic potential. 

### 3.5. Overexpression of *BMP4* Improved Chondro- and Osteogenesis *In Vitro* but Reduced the Ability to Form Adipocytes

**Figure 5:**
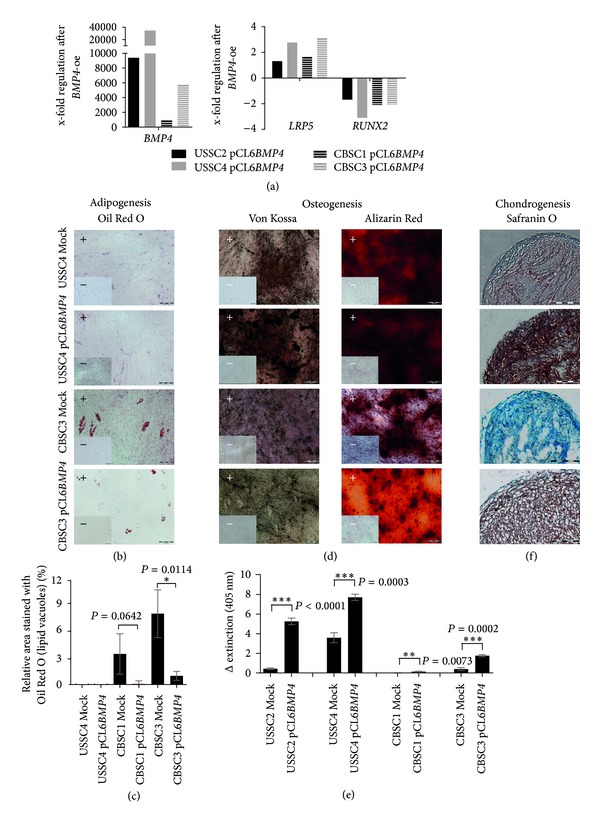
Lentiviral overexpression of *BMP4* in two USSC and two CBSC cell lines. With regard to the staining, only one representative cell line per cell type is shown. (a) Quantitative RT-PCR analysis of gene expression (in undifferentiated cells) after overexpression in relation to the control cells (Mock). *RPL13A* was used as housekeeping gene. (b): Representative illustration of the Oil Red O staining after adipogenic differentiation (21 d) of *BMP4-*transfected and control-cells. +: induced cells; −: non-induced cells. Lipid vacuoles are stained red. Scale bar: 100 *μ*m. (c) ImageJ-based quantification of Oil Red O staining. *P = 0.01 to 0.05, significant (unpaired *t*-test). (d) Osteogenic differentiation experiments in overexpressed and Mock cells. After 14 days of differentiation, Von Kossa and Alizarin Red S staining were performed in the induced (+) and non-induced (−) cells. Mineralized areas are stained in brown/black or red, respectively. Scale bar: 200 *μ*m. (e) Subsequent quantification of the bound Alizarin Red S-dye. **P = 0.001 to 0.005, very significant; ***P < 0,001, extremely significant (unpaired *t*-test). (f) Safranin O staining of transfected and control cells after chondrogenic differentiation in pellet culture for 21 days. Proteoglycans are stained purple/red. Scale bar: 200 *μ*m. *BMP4: *bone morphogenetic protein 4, *LRP5*: low-density lipoprotein receptor-related protein 5, *RUNX2: *runt-related transcription factor 2, and *RPL13A*: ribosomal protein L13A.

The bone morphogenetic protein 4 (*BMP4*) was strongly expressed in BMSC, while the CB-derived cell populations exhibited a weaker expression (Figure 2). The lentiviral gene delivery of *BMP4* was accomplished in two USSC and two CBSC cell lines as proved by qRT-PCR (Figure 5(a), USSC2: 9381-fold stronger as Mock-cells, USSC4: 34598-fold stronger, CBSC1: 890-fold stronger, and CBSC3: 5714-fold stronger). The overexpression caused a reduced expression of *RUNX2* while *LRP5* (low-density lipoprotein receptor-related protein 5), which is associated with the WNT signaling pathway, showed an upregulation (Figure 5(a), *RUNX2*: USSC2: −1.7-fold compared to Mock cells, USSC4: −3.1-fold, CBSC1: −2.1-fold, CBSC3: −2.1-fold; *LRP5*: USSC2: 1.3-fold, USSC4: 2.8-fold, CBSC1: 1.6-fold, and CBSC3: 3.1-fold). The influence on the potential to differentiate into adipocytes, osteoblasts, or chondrocytes was assed via *in vitro* differentiation assays. The lack of the ability of USSC to differentiate into adipocytes [5] was not influenced by the overexpression of *BMP4*. On the contrary, the potential of CBSC to differentiate towards the adipogenic lineage was diminished after overexpression of *BMP4,* as assessed by Oil Red O staining and ImageJ-based quantification of the staining (Figures 5(b) and 5(c), unpaired *t*-test: CBSC1 Mock/pCL6*BMP4*, *P* = 0.06; CBSC3 Mock/pCL6*BMP4*, *P* = 0.01). *BMP4*-transfected USSC and CBSC secreted more proteoglycans during chondrogenic differentiation in pellet culture illustrated by the intensified purple/red Safranin O staining (Figure 5(f)). Likewise, during osteogenic differentiation, transfected cells showed an enhanced calcification which was proved by Von Kossa and Alizarin Red S staining with subsequent quantification (Figures 5(d) and 5(e), USSC2: *P* < 0.0001, extremely significant; USSC4: *P* = 0.0003, extremely significant; CBSC1: *P* = 0.0073, very significant; CBSC3: *P* = 0.0002, extremely significant). 

In summary, the overexpression of *BMP4* in two USSC and two CBSC cell lines resulted in a “switch” of the cell capability: the adipogenic differentiation potential was reduced, while the chondrogenic and osteogenic potentials were improved.

## 4. Discussion

The evaluation of the multilineage *in vivo* differentiation capacity of distinct stromal cell types is of particular importance, for example, in the field of bone tissue engineering regarding the applied cell source. Basis for these *in vivo* assays is the detailed analysis of the gene expression profile in combination with the *in vitro* differentiation potential to gain insight into the inherent capacity of a stromal cell and to define genes relevant to the regulation of differentiation. Therefore, microarray gene expression analyses were performed in the present study to compare stromal cells from bone marrow (BMSC) and cord blood (USSC and CBSC). In a second step, genes expressed differentially in the cell populations were validated by quantitative RT-PCR analyses. Finally, three genes associated with cartilage/bone-formation (*BSP, OSX,* and* BMP4*) were overexpressed to assess their function in cord blood-derived stromal cells.

Microarray analyses (Figure 1) revealed a closer related gene expression pattern of USSC and CBSC reflecting their common cord blood origin. In contrast to the CB-derived cell types, the three BMSC cell lines revealed a higher variance in their expression profile (Figure 1(b)) which may be due to variances regarding donor age or sex. An “osteogenic signature” was determined for BMSC following microarray and qRT-PCR analyses (Figures 1 and 2) according to the *in vivo* role of these cells creating the endosteal niche which regulates the self-renewal and differentiation of hematopoietic stem cells [23]. In accordance with this biological function, BMSC expressed high levels of *PITX2* (Figure 2) which—beside other functions—were described to affect the hematopoietic supportive capacity of bone marrow stromal cells [24]. The osteogenesis-related genes *BSP*, *OSX*, *BMP4,* and *OC* revealed a stronger expression in BMSC compared to USSC and CBSC (Figure 2) which reflects the more immature status of the neonatal cord blood-derived stromal cells. Unlike BMSC, USSC and CBSC had a reduced predisposition towards skeletal development.

The phosphoprotein *BSP* is part of the human bone extracellular matrix [14]. *Bsp* expression has been described to be essential for the *in vivo* bone forming potential of murine BMSC [15]. *Bsp* knockout mice (*BSP* ^−/−^) displayed an impaired bone growth and mineralization associated with reduced bone formation [25]. In the present study, *BSP* was described as a gene expressed discriminatively between BMSC and the CB-derived cell types (Figure 2). *BSP* can be regarded as potential key player in the regulation of bone formation which was confirmed by the enhanced calcification of *BSP* overexpressing USSC in an *in vitro* osteogenesis assay (Figure 3). Furthermore, the increase in calcification corresponds to the study by Hunter and Goldberg, reporting that *BSP* initiates hydroxyapatite crystal formation during bone formation [26]. 

In contrast to *BSP*, the overexpression of the transcription factor *OSX* (*SP7*) in USSC resulted in a decreased *in vitro* osteogenic capacity (Figure 4), although *OSX* is commonly described as an essential regulator for bone formation [13]. *Osx* null mice display normal cartilage with mature hypertrophic chondrocytes but fail to form bone which highlights the specific role for *Osx* in the differentiation of osteoblasts [13, 27]. In the study herein, the overexpression of *OSX *caused a downregulation of upstream-located *RUNX2 *in a negative feedback loop (Figure 4(a)). The enhanced expression of *BSP* and *BMP2* after transfection of *OSX* into USSC (Figure 4(a)) did not support the mineralization. Comparable results were presented in a study by Kurata et al. in human primary fetal stromal cells. Overexpression of *OSX* did not result in extracellular calcium crystals [28]. Likewise, Yoshida and coworkers overexpressed *Osx* in murine primary osteoblasts and reported a reduced mineralization at a late stage of osteoblast differentiation. Furthermore, *Osx* transgenic mice exhibited a reduced bone mineral density (osteopenia) [29]. In contrast, other overexpression studies in murine-adipose-tissue-derived stromal cells [30] or murine BMSC [31] reported an improved osteogenic potential. These controversial results may be due to the different cell types used for overexpression experiments which potentially reveal a distinct expression pattern of cofactors required to induce differentiation. One of these is *NFAT* (nuclear factor of activated T cells) which forms a complex with *OSX* to control osteoblastic bone formation [32]. 

*BMP4* is a secreted signaling molecule that plays an essential role during embryogenesis [9, 33]. Regarding limb development, a threshold level of BMP signaling is required for early chondrogenic processes. The loss of both *Bmp2* and *Bmp4* during murine knockout experiments resulted in impaired osteogenesis [11]. In the present study, overexpression of *BMP4* supported the chondro- and osteogenic but reduced the adipogenic differentiation of human cells (Figure 5) which corresponds to previous studies in mice. Kan et al. described *Bmp4* transgenic mice which developed a phenotype characterized by progressive heterotopic bone formation [34]. Another report by Duprez and colleagues addressed the influence of ectopic retroviral overexpression of *BMP4* in developing chick limbs. The overexpression resulted in an increase in the volume of cartilage elements caused by an extended amount of matrix [35]. In the study herein, *BMP4* overexpression in human neonatal cells led to a reduced expression of *RUNX2* (Figure 5(a)) which suggests a *RUNX2*-independent stimulation of osteogenic differentiation. In contrast, the expression of *LRP5*, which functions as coreceptor of the Frizzled (Fzd)-receptors during canonical WNT signaling, was enhanced after *BMP4* overexpression (Figure 5(a)), indicating a role of WNT signaling in the *BMP4*-caused promotion of mineralization. Due to the fact that osteoblasts and adipocytes share a common progenitor, some differentiation factors, such as *PPAR*γ**(peroxisome proliferator-activated receptor gamma), are essential not only in the cell fate decision by induction of adipogenesis but also in suppression of osteogenic developmental processes [36, 37] or vice versa. The interpretation of the data demonstrated in the present paper indicates a potential role of *BMP4* in the cell fate decision of precursor cells promoting the differentiation towards the chondro-/osteogenic direction and suppressing the adipogenic differentiation.

In summary, the overexpression experiments suggested a supportive role for *BSP* and *BMP4* during *in vitro* osteoblast differentiation; *BMP4* additionally promoted the chondrogenic but diminished the adipogenic differentiation potential which indicates a role for *BMP4* in the cell fate determination towards the osteoblast lineage. In contrast, *OSX*-overexpression did not support mineralization.

Beside the *DLK1*- and *HOX*-gene expressions described by our group in former studies [5, 7], *MSX2* is a further gene expressed differently between USSC and CBSC (Figure 2). The expression was significantly stronger in USSC compared to CBSC. Mice deficient in the homeobox gene *MSX2* displayed defects in endochondral bone formation [38]. Ichida and colleagues reported a promotive effect of *MSX2 *on the differentiation of mesenchymal cells towards osteoblasts. In contrast, *MSX2* inhibited the expression of *PPAR*γ** resulting in a diminished adipogenic differentiation potential [39]. These data are consistent with our results reporting that USSC, which exhibit a strong osteogenic potential *in vitro* but are not able to differentiate into adipocytes, exhibited a high expression level of *MSX2*. In contrast, CBSC, which have a diminished potential to differentiate towards the osteogenic lineage *in vitro* but are able to form adipocytes, did not express *MSX2* (Figures 2–5 and [4]). 

Contrary to *MSX2*, both CB-derived cell types strongly expressed *MYC* while BMSC exhibited only a slight expression (Figure 2). *MYC* encodes the transcription factor C-MYC which activates or represses genes involved in cell growth or cell cycle control. For example, C-MYC represses the expression of the growth arrest gene *GAS1,* hereby promoting cell proliferation [40]. This correlates with the extended growth potential of USSC and CBSC compared to the reduced growth potential of BMSC [4]. 

## 5. Conclusions

In contrast to bone-marrow-derived stromal cells, the cord blood-derived cell types USSC and CBSC lacked a signature related to skeletal development as shown by microarray gene expression and quantitative RT-PCR analyses. After overexpression experiments,* BSP* and *BMP4*, which were absent in the CB-derived cells, were defined as potential key players affecting the differentiation potential. *BSP* influenced the calcification during osteogenic differentiation assays. *BMP4* reduced the adipogenic potential but enhanced the secretion of proteoglycans during chondrogenic as well as the calcification after osteogenic differentiation assays. Thus, *BMP4* seems to determine the cell fate towards the chondro-/osteogenic lineage. 

Understanding the influence of different signaling pathways that control differentiation is essential to predict the applicability of a distinct cell population for regenerative therapy. Hence, BMSC seem to be a cell source more suitable for bone tissue engineering approaches compared to USSC or CBSC which exhibited a more immature signature.

## Supplementary Material

Table S1. Primer sequences. For PITX2 and MYC, please see materials and methods section. Figure S1. pCL6IEGwo plasmid used for lentiviral overexpression experiments. Figure S2. PCL6IEGwo including the inserted genes (BSP, OSX and BMP4).

## Figures and Tables

**Table 1:** Osteogenesis-related genes expressed uniquely in USSC, CBSC, or BMSC. After Venn diagram analyses (Figure 1(a)), the genes expressed uniquely in one cell population (USSC: 304, CBSC: 251, and BMSC: 375) were assigned to gene ontology (GO) terms using the “Functional Annotation Cluster Tool” provided by DAVID Bioinformatics Resources (http://david.abcc.ncifcrf.gov/). Only those GO terms associated with “bone formation” are presented.

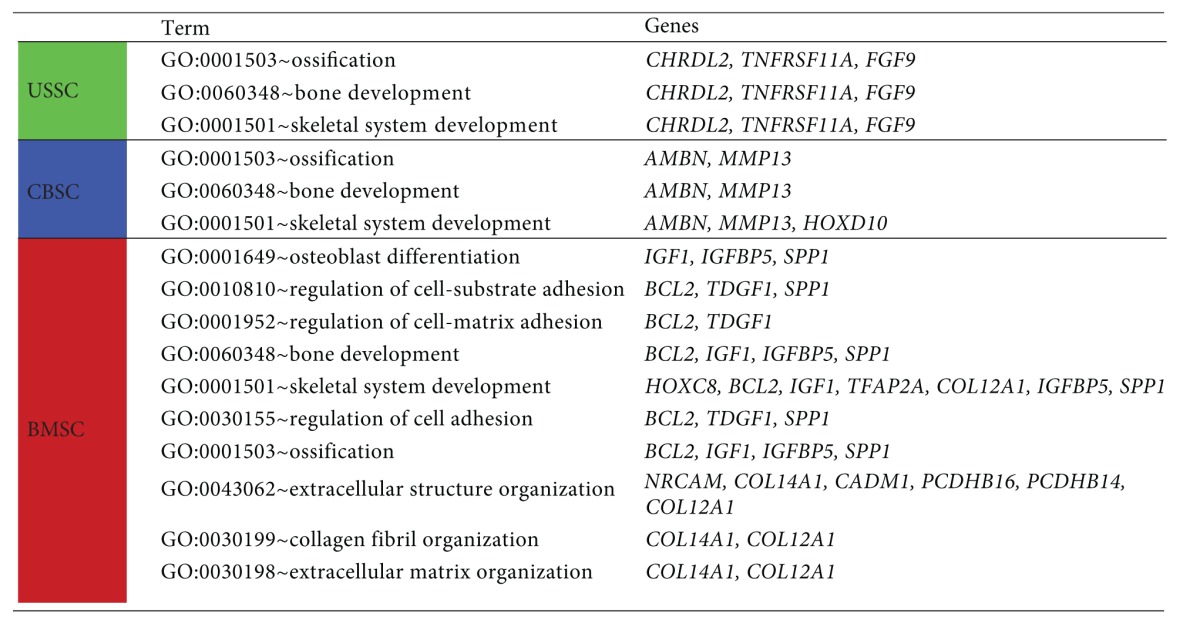

*AMBN*: ameloblastin (enamel matrix protein);* BCL2*: B-cell CLL/lymphoma 2; *CADM1*: cell adhesion molecule 1; *CHRDL2*: chordin-like 2; *COL12A1/14A1*: collagen type XII alpha 1/type XIV alpha 1; *FGF9*: fibroblast growth factor 9; *HOXC8/D10*: homeobox C8/D10; *IGF1*: insulin-like growth factor 1; *IGFBP5*: insulin-like growth factor binding protein 5; *MMP13*: matrix metalloproteinase 13; *NRCAM*: neuronal cell adhesion molecule; *PCDHB14/16*: protocadherin beta 14/16; *SPP1*: secreted phosphoprotein 1 (Osteopontin); *TDGF1*: teratocarcinoma-derived growth factor 1; *TFAP2A*: transcription factor AP-2 alpha; *TNFRSF11A*: tumor necrosis factor receptor superfamily, member 11a.
