# Supplementary material for: Comparing the Gene Expression Profile of Stromal Cells from Human Cord Blood and Bone Marrow: Lack of the Typical “Bone” Signature in Cord Blood Cells
Source: Stem Cells Int. 2013 Sep 16;2013:631984. doi: 10.1155/2013/631984 (PMC3791663; doi:10.1155/2013/631984)
Supplement: Supplementary file 1 — Table S1. Primer sequences. For PITX2 and MYC, please see materials and methods section. Figure S1. pCL6IEGwo plasmid used for lentiviral overexpression experiments. Figure S2. PCL6IEGwo including the inserted genes (BSP, OSX and BMP4). [file 631984.f1.ppt]

## Slide 1
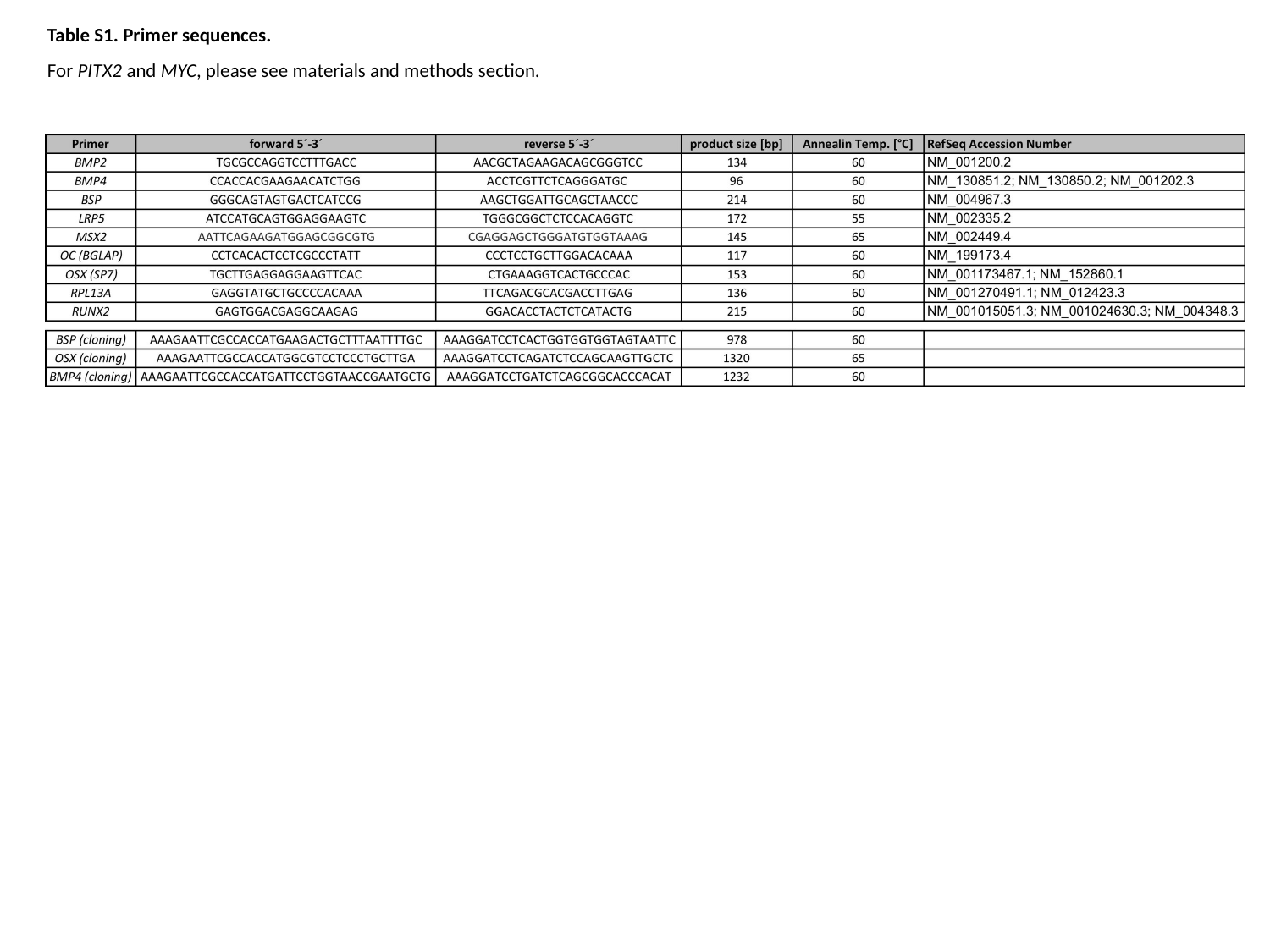

Table S1. Primer sequences.
For PITX2 and MYC, please see materials and methods section.

## Slide 2
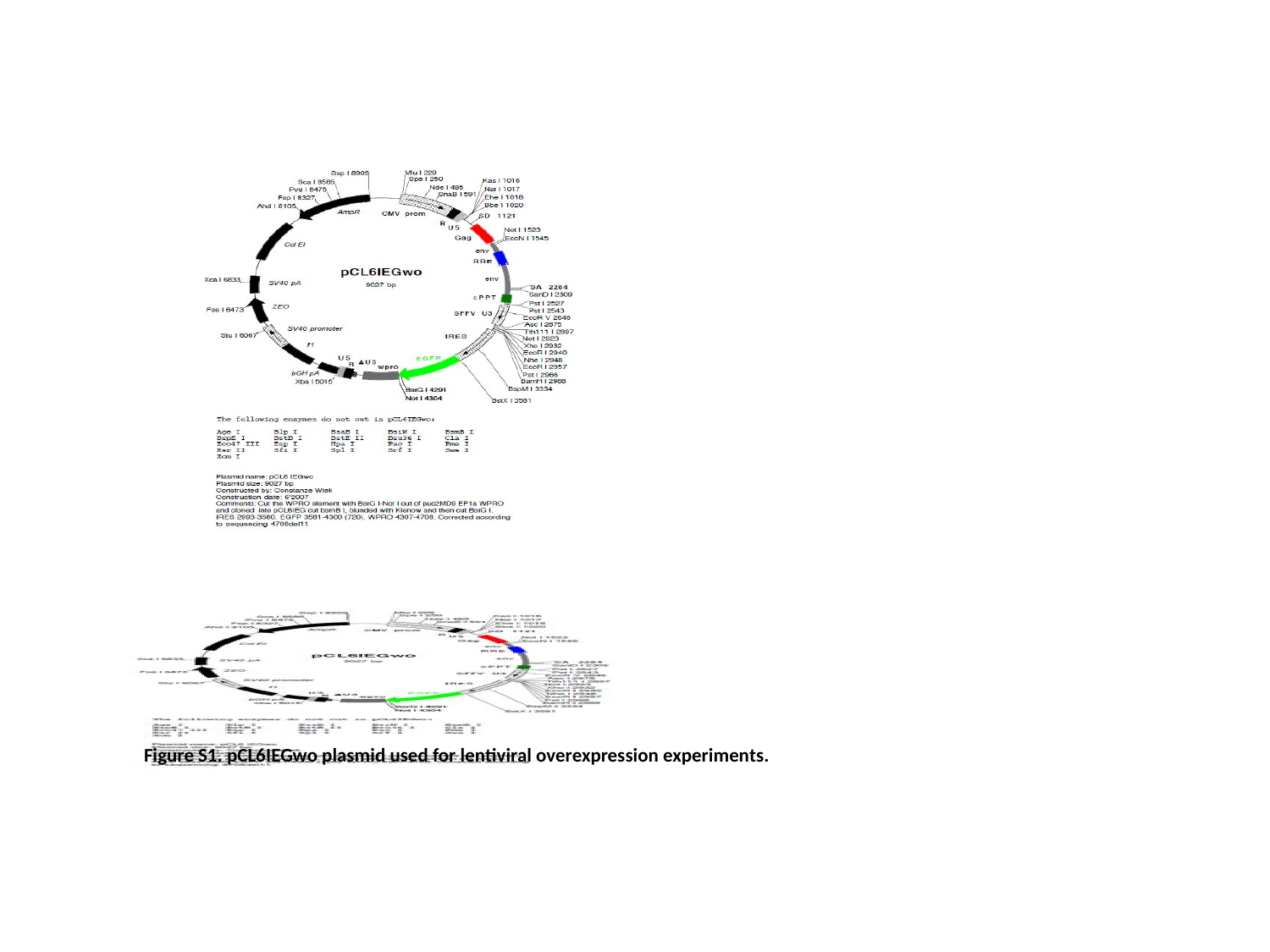

Figure S1. pCL6IEGwo plasmid used for lentiviral overexpression experiments.

## Slide 3
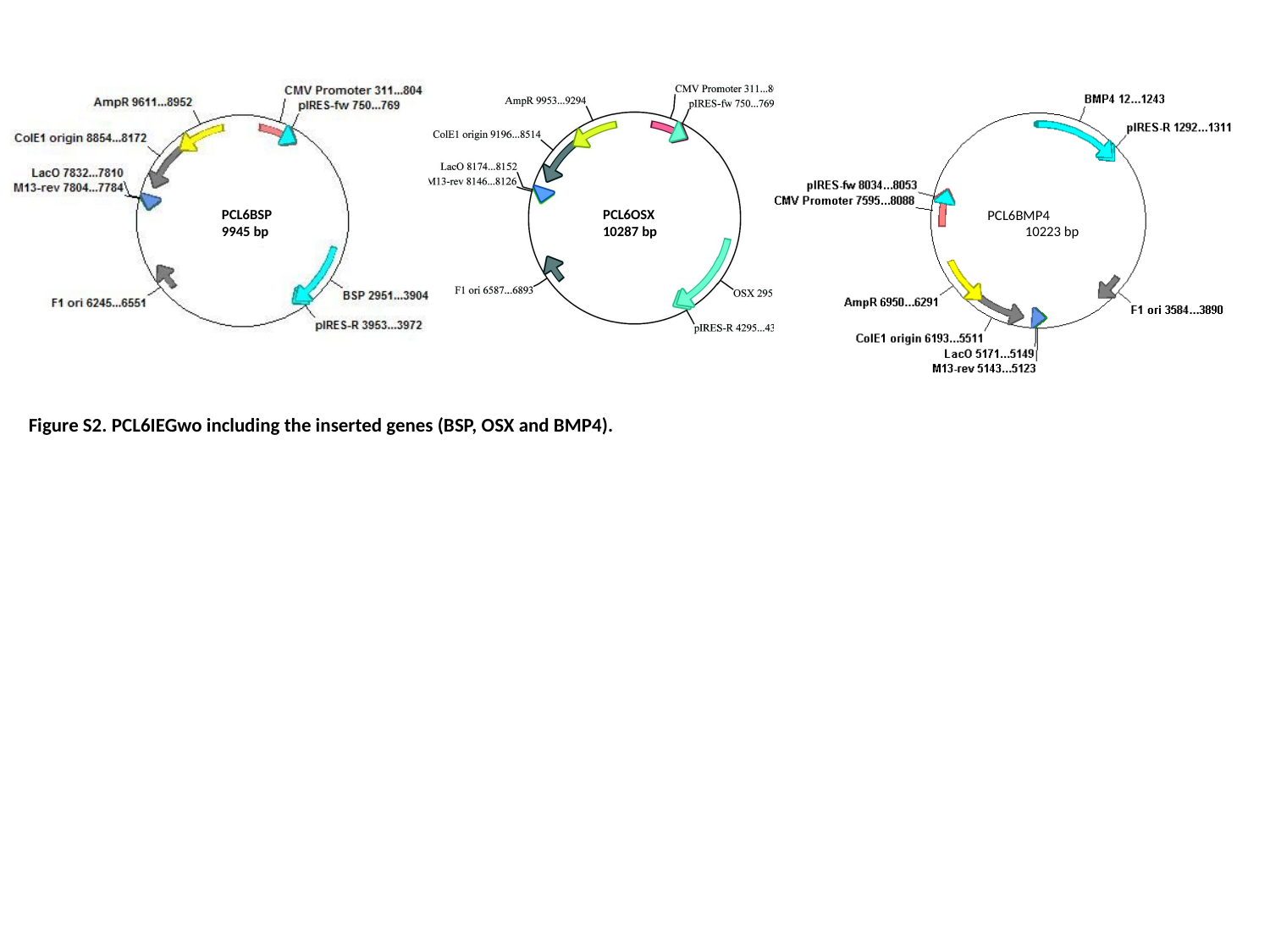

PCL6BSP 9945 bp
PCL6OSX 10287 bp
PCL6BMP4 10223 bp
Figure S2. PCL6IEGwo including the inserted genes (BSP, OSX and BMP4).
